# Licensed Homes for Inebriates

**Published:** 1913-12-06

**Authors:** Beatrice B. Temple, Emily Wilberforce, Adela de C. Pereira

**Affiliations:** Chairman, C.E.T.S. Women's Union. 4 Sanctuary, Westminster, S.W.; Chairman, C.E.T.S. Women's Union. 4 Sanctuary, Westminster, S.W.; Chairman, C.E.T.S. Women's Union. 4 Sanctuary, Westminster, S.W.


*To the Editor of* The Hospital.

### Licensed Homes for Inebriates.

*To the Editor of* The Hospital.

Sir,—May we beg to put before your readers a matter of great importance? From time to time there appear in the press reports of utterances by public men in which institutions which are somewhat loosely described as "inebriate homes" are condemned without qualification. The general effect of such remarks is to give the public an erroneous impression that all homes for the reformation of the intemperate are failures. To those who are acquainted with the subject of inebriety and its treatment it is well known that even the State reformatory, which is in the minds of those who speak disparagingly of the work of homes for inebriates, does, in fact, perform a thoroughly useful function. In this letter we are not concerned to defend reformatories, though, in passing, we would remind your readers that if they have effected nothing else they do keep out of harm's way a large number of the most desperate cases for a certain period. Apart from which it is a mere matter of fact that a certain steady, if small, percentage of those passing through the reformatories do not relapse into intemperate habits.

The main object of our present communication, however, is to mention the very successful work of licensed retreats or homes for inebriate women and men, and especially to draw attention to those which the Church of England Temperance Society has for twenty-five years carried on with great benefit to a very large number of victims. We have at present four excellent homes for women, situated respectively in the suburbs of London, in Norfolk, on the outskirts of Birmingham, and at Torquay. In these institutions we receive women of all social classes at fees varying from 6s. to £1 1s. weekly. No patient is accepted for less than twelve months, which is the result of our long experience of the work. The treatment is that of a home, and not a house of detention. First and always we rely upon the spiritual influence to effect a "cure." In addition, kind and sympathetic treatment, good food, fresh air, occupation, amusement, and enforced abstinence are employed. The result is that of the many hundreds who have passed through our hands a large proportion have stood firm and have become useful, happy members of society as the result of their sojourn in our homes. Experience teaches us that in spite of all our efforts this work and its success are all too little known by the general public, and we shall feel deeply grateful to you if you will give publicity to this letter at a time when the treatment and detention of inebriates is very much in the minds of thoughtful people. Our Secretary, the Rev. Gerald A. Thompson, 4 The Sanctuary, Westminster, will be happy to send any further particulars to those of your readers who desire them. While we have confined ourselves in this letter to the matter of the homes for women, we should like to add that our Society has also a large establishment—under the care of a clergyman, who is also a doctor of medicine—for the reception of men who have fallen into intemperance.—Yours very truly,

Beatrice B. Temple,
Emily Wilberforce,
Adela de C. Pereira,
Chairman, C.E.T.S. Women's Union.

4 Sanctuary, Westminster, S.W.

